# Olfactory preference conditioning changes the reward value of reinforced and non-reinforced odors

**DOI:** 10.3389/fnbeh.2014.00229

**Published:** 2014-07-03

**Authors:** Nicolas Torquet, Pascaline Aimé, Belkacem Messaoudi, Samuel Garcia, Elodie Ey, Rémi Gervais, A. Karyn Julliard, Nadine Ravel

**Affiliations:** ^1^Neurophysiology and Behavior Laboratory, Neuroscience Paris-Seine – IBPS, CNRS UMR 8246 – UPMC UM CR18 – INSERM U1130Paris, France; ^2^Department of Pathology and Cell Biology, Columbia University Medical CenterNew York, USA; ^3^Equipe Olfaction: du Codage à la Mémoire, Centre de Recherche en Neurosciences de Lyon, CNRS UMR 5292 - INSERM U1028, Université Lyon 1Lyon, France; ^4^Human Genetics and Cognitive Functions, Neuroscience Department, Institut Pasteur, CNRS UMR 3571 Genes, Synapses and CognitionParis, France

**Keywords:** olfaction, odor, behavior, rat, olfactory preference, conditioning, discrimination

## Abstract

Olfaction is determinant for the organization of rodent behavior. In a feeding context, rodents must quickly discriminate whether a nutrient can be ingested or whether it represents a potential danger to them. To understand the learning processes that support food choice, aversive olfactory learning and flavor appetitive learning have been extensively studied. In contrast, little is currently known about olfactory appetitive learning and its mechanisms. We designed a new paradigm to study conditioned olfactory preference in rats. After 8 days of exposure to a pair of odors (one paired with sucrose and the other with water), rats developed a strong and stable preference for the odor associated with the sucrose solution. A series of experiments were conducted to further analyze changes in reward value induced by this paradigm for both stimuli. As expected, the reward value of the reinforced odor changed positively. Interestingly, the reward value of the alternative odor decreased. This devaluation had an impact on further odor comparisons that the animal had to make. This result suggests that appetitive conditioning involving a comparison between two odors not only leads to a change in the reward value of the reinforced odor, but also induces a stable devaluation of the non-reinforced stimulus.

## Introduction

Rodents are macrosmatic animals with olfactory abilities allowing them to detect and discriminate a wide range of odors and accomplish complex olfactory-mediated cognitive tasks (Slotnick, 2001). By relying on olfaction, rodents can learn to discriminate distant appetitive and edible items from aversive and potentially hazardous items. Although some odors or tastes can generate innate aversion as well as preference behaviors (Apfelbach et al., 2005; Kobayakawa et al., 2007; Ventura and Worobey, 2013), most food choices result from an associative process between food sensory characteristics and the post-ingestive consequences. This process most often leads to switch the initial neutral reward value of a specific food toward either a preference or an aversion (Slotnick, 2001; Gautam and Verhagen, 2010).

When consuming, we are exposed to two types of chemosensory stimuli: first, the odor, when items are at distance, and then the flavor (a combination of retronasally transmitted olfactory stimulation and taste) when items are in the mouth (Pierce and Halpern, 1996). These odor-taste interactions have been described as taste-odor synesthesia resulting from pairing of the two chemosensory modalities via oral and retronasal stimulation during food ingestion (Verhagen and Engelen, 2006). Indeed, in humans, food-related odorants are often reported and described as tastes (vanilla for example reported as sweet) and a novel odor can acquire a taste even after a single pairing (for a review see Stevenson and Tomiczek, 2007). From experimental work in rodent, it has become clear that taste-like qualities of odors are learned and are not innate (Gautam and Verhagen, 2010).

A large number of studies propose an unraveling of the contribution of each type of chemosensory stimulus in the learning process, most of them using aversive conditioning paradigms (Palmerino et al., 1980; Batsell et al., 1999; Batsell and Blankenship, 2002; Miranda, 2012). In such protocols, the presentation of a solution characterized by a specific odor or taste can be associated with a gastric malaise induced by an intraperitoneal injection of lithium chloride and leads to a strong further avoidance of this solution (Garcia et al., 1968). Interestingly, whereas a pure taste aversion could be induced, even when the malaise is delayed from the ingestion, odor aversion alone is more difficult to obtain and less resistant to an increase of ingestion-malaise delay (Palmerino et al., 1980). However, when the odor is combined with a specific taste during the initial experience (taste potentiated odor aversion, TPOA) or ingested without any gustatory impact, a very robust aversive behavior is obtained even when the odor is presented alone during the test (Rusiniak et al., 1979; Palmerino et al., 1980; Miranda, 2012). As a consequence, further animal models using conditioned flavor aversion or preference were developed. These models showed that an odor present in a solution or an ingested aliment could become aversive (Slotnick et al., 1997; Chapuis et al., 2007) or attractive exactly as observed in taste models (Holder, 1991; Boakes et al., 2007).

The respective role of the individual components of a flavor, odor and taste, have been less explored regarding appetitive learning. To our knowledge, only two paradigms have clearly established a strict Conditioned Olfactory Preference (COP; Holder, 1991; Lucas and Sclafani, 1995). Compared to previous work in which odorants were consumed by the animal, in the Holder's paradigm, the odors used (almond or peppermint) were presented during 4 consecutive days in close proximity with the ingested solution (sucrose or saccharin) but the rats never made gustatory contact with them. A two-bottle test performed 24 h later revealed that animals preferred the odor previously paired with sucrose over saccharin, suggesting both a calorie-mediated and an appetitive taste-mediated preference (Holder, 1991). In this paradigm however, as each odor is paired with a sugar-sweetened solution, it is difficult to interpret the real change of the reward value for the odor paired with non-nutritive saccharin. Indeed previous studies have demonstrated that the animals preferred odor paired with the nutritive diet (Baker and Booth, 1989; Holder, 1991) and strong odor preferences could be obtained for odors paired with the post ingestive actions of nutrients without the presence of added taste cues (Lucas and Sclafani, 1995). In the present study, we therefore propose to investigate the influence of appetitive learning on the reward value of both the odor used as the conditioned stimulus (CS+), paired with sucrose, and a second odor (CS−) that is paired with the neutral solution, pure water. Additionally, we also investigate whether COP could be obtained from an initially preferred odor as well as from a less preferred one.

Compared to previous studies (Holder, 1991; Lucas and Sclafani, 1995), the present work provides new and additional informations on Conditioned Olfactory Preference. In experiment 1, we first evaluated the spontaneous behavioral preferences of several odors presented in pairs. We then induced a conditioned olfactory preference by coupling one of the odors with a sucrose solution, and a second one with pure water, which remains a positive stimulus for water-deprived animals but less appetitive. The protocol efficacy was measured immediately and 1 month later by testing how the conditioning procedure impacts the initial observed preference for the conditioning pair of odors. A series of additional experiments was performed to evaluate how the conditioning procedure also affected the spontaneous preference for the two tested odors compared to others not involved in the conditioning paradigm (Experiments 2 and 3). This allowed us to demonstrate that, as expected, the conditioned odor was reinforced (increased positive valence). Interestingly, we also reported that the reward value of the non-conditioned odor was depreciated compared to any non-training odor.

## Materials and methods

### Animals

Experiments were carried out in accordance with the European Community Council Directive of November 24th, 1986 (86/609/EEC) for the care and use of laboratory animals. The experimental protocols were approved by the Lyon1 University Ethics Committee (Direction of Veterinary Service #693870202).

A total of forty male Wistar rats (275–300 g, Charles River, L'arbresle, France) were involved in this study. They were housed individually in Plexiglas chambers at constant temperature and relative humidity (22 ± 0.5°C and 50 ± 5%) and under a 12-h light: 12-h dark cycle (light on at 6.00 AM) at least 1 week before the beginning of the experiments.

A total of forty rats were included in the 3 experiments. Sixteen of them participated in experiments 1 and 2. In experiment 1, eight rats were assigned to either group 1 or group 2. In experiment 2, animals from both groups were equally distributed between group 3 and group 4. Twenty-four naïve animals participated in experiment 3.

### Odors

Odors were purchased from Sigma-Aldrich (France). According to their chemical properties, they were diluted either in mineral oil or in water. The dilution was also adjusted as a function of their vapor pressure to be judged as moderate and equal in intensity by the experimentalist. In the following, + and − symbols with brackets [] refer to the enantiomer structure of the odors while the + and − symbols outside brackets refers to the association made during conditioning with sucrose (+suc) or water (−) respectively. Geraniol, eugenol, and two enantiomers of carvone (carvone[−] and carvone[+]) were diluted to 10% in mineral oil, limonene[−] was diluted to 5% in mineral oil and iso-amylacetate was used at a dilution of 10% in water. Geraniol and eugenol were used for experiment 1, carvone[−], carvone[+] and limonene[−] for experiment 2 and the two enantiomers of carvone, limonene [−] and iso-amylacetate in experiment 3. 150 μL of each odor solution was distributed on a 3-cm cotton disc (3M™ T156 Oil Sorbent Sheets). The disc was placed into the 3.2-cm cap of a tube, face turned toward the cage and secured in place by a thin grid of metal. The metal grid, the disc and the 3-cm cap were punched with a 16 mm hole allowing them to be placed around the spout of the bottle of drinking solution. Therefore, the rats could never touch the disc, as described in the experiments of Holder (1991). Importantly, such a device also precludes the bottle solution to be polluted by the odor.

### Experimental set up (Figure 1)

Behavioral tests were conducted in parallel for four rats in individual Plexiglas operant chambers (330 × 210 × 180 mm). The chambers were set side by side so that the animals could not see each other. Two plastic tubes were mounted on the opposite sides of the flat ceiling of each chamber. These tubes (made from 15 ml polypropylene conical centrifuge tubes, Falcon, France) were cut and fire polished to build a 0.4 mm spout which protruded approximately 5 mm into the chamber, allowing the rats to drink from the spout with ease by rising up. The amount of liquid consumed by the rat from each tube was measured by a device called licking box. This system has already been used to measure odor detection in different states of satiety and described in details elsewhere (Aimé et al., 2007). Each tube was connected to a custom-made capacitance circuit which allowed detecting, visualizing and recording of individual licks at each bottle across time during the experimental sessions. Generated files were then transferred into a database developed in our laboratory to be analyzed offline. In parallel, the global consumption of each rat from each bottle was also measured to check the accuracy of the licking system.

**Figure 1:**
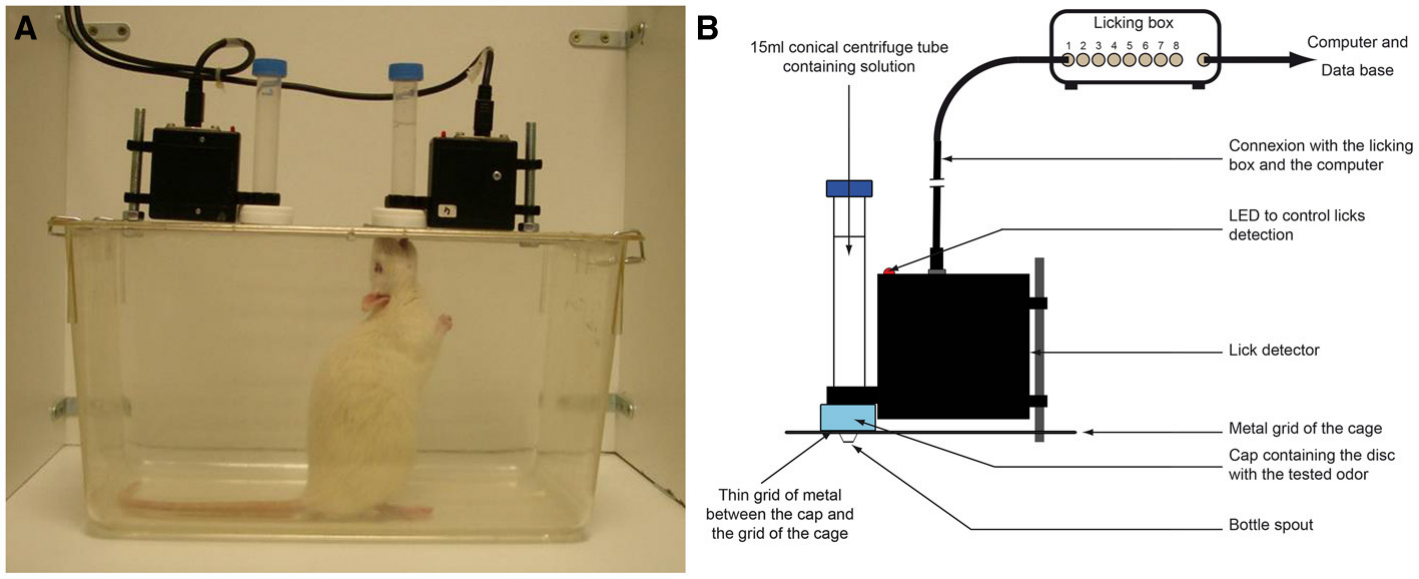
**Experimental Set-up. (A)** Experimental set up. When the rat drinks, its nose is in close contact with the odor to ensure a good association with the drinking solution. The odor is never ingested. **(B)** Detail of the bottle and the licking box system. The tube containing the solution is coupled to a lick detector. The odor is placed on a disk into a cap face turned toward the cage. A thin grid of metal between the cap and the grid of the cage prevents the rat to lick the disk. Each lick at the bottle's spout is detected via the licking box and sent to a computer to be recorded into a database. A homemade computer interface allows to display the recorded licks for each bottle.

### General protocol

To motivate their drinking behavior, rats had been acclimated to a restricted access to water during 1 week before the beginning of habituation. During the overall course of the study, their daily access to water was restricted to 15 min in the experimental cage, supplemented by a 20 min period of free access each afternoon at 6:00 PM in their home cages.

#### Habituation

During 5 days, rats were placed in the experimental cages with two bottles of water for a 15 min session. This phase was devoted to train rats to drink from the device, and to avoid stress during the conditioning.

#### Pretest

To assess a possible spontaneous aversion or preference, each pair of odors to be used in the following experiment was presented to the animal. During this phase, for each 15 min session, two bottles of tap water were each associated with one odor of the pair and simultaneously presented. The same pair was tested on two consecutive days switching the location of the odors to rule out place preference.

#### Conditioning

During the 8 days of conditioning, rats were exposed during 15 min to two odors paired with two bottles containing different solutions. Odor A was paired with a bottle containing a solution of 3.4% sucrose (A+suc), while odor B was paired with a bottle of tap water (B−). The preference for specific sugar concentration varies according to the level of water deprivation imposed to the animals. We used a concentration of 3.4% of sucrose as validated by Holder (1991). In our water restriction condition, this concentration was still preferred by the rats over water. Throughout the study, the position of each bottle was switched each day to rule out place preference.

#### Final test

Two bottles containing tap water associated with odor A or B were presented for 15 min on two consecutive daily sessions switching their position. Results are presented as the mean intake over 2 days allowing for position switch.

The same protocol was used for the three experiments, with a variation in conditioning odors according to the experiment (see Table 1 for the details of each group). When additional tests were performed (experiments 2 and 3), two additional conditioning sessions were conducted between each test to avoid a possible extinction of preference induced by the repetition of odor presentation without sucrose-pairing.

**Table 1:** **Summary of experiments**.

	**Animals**	**Pretests**			**Conditioning**	**First test**	**Second test**	**Third test**	**LTM test**
**Exp. 1**	Group 1	G/E			G+suc	G+/E−			G+/E−
	*n* = 8				/				
					E−				
	Group 2	E/G			E+suc	E+/G−			E+/G−
	*n* = 8				/				
					G−				
**Exp. 2**	Group 3	C[−]	C[+]	C[−]	C[−]+suc	C[−]+	C[+]	C[−]+	
	*n* = 8	/	/	/	/	/	/	/	
		C[+]	L[−]	L[−]	L[−]−	L[−]−	L[−]−	C[+]	
	Group 4						C[−]+	C[+]	
	*n* = 8						/	/	
							C[+]	L[−]−	
**Exp. 3**	Group 1	C[−]	L[−]	C[−]	C[−]+suc	C[−]+	L[−]−	C[−]+	
	*n* = 12	/	/	/	/	/	/	/	
		G	I	L[−]	L[−]−	L[−]−	I	G	
	Group 2		C[+]				C[+]		
	*n* = 12		/				/		
			E				E		

*Pretest, Odors presented associated with water; Conditioning, +suc, odor paired with sucrose (CS+); −, odor paired with water (CS−); Tests, +, odor previously used as a CS+; −, odor previously used as a CS−; no sign, this odor has not been included in the conditioning*.

### Statistical analysis

The coefficient of correlation between the number of licks and the global water consumption measured during experiment 1 indicated a good reliability of our licking system (*r* = 0.985). As a consequence, we chose the number of licks as the study variable. To normalize data, we expressed consumption from each bottle as a ratio of the number of licks for this bottle on the sum of the number of licks for the two bottles. This variable was then compared between subjects according to the different experimental groups and for a given subject as a function of experimental condition (pretest, test). Within group differences were then analyzed using the non-parametric Wilcoxon test (WT). The changes in bottle position were never found to induce a significant difference in liquid consumption, we therefore pooled the average number of licks on each bottle at the two positions.

## Results

### Experiment 1

#### Specific methods

Group 1 (*n* = 8) was conditioned to geraniol [i.e., geraniol was paired with sucrose (G+suc) and eugenol with water (E−)] while group 2 (*n* = 8) was conditioned to eugenol [i.e., eugenol was paired with sucrose (E+suc) and geraniol with water (G−)]. Two tests were performed to estimate olfactory preference: one immediately after the end of the conditioning period and the other, 1 month later to investigate the long-term retention of conditioning (see Table 1).

#### Results (Figure 2)

**Figure 2:**
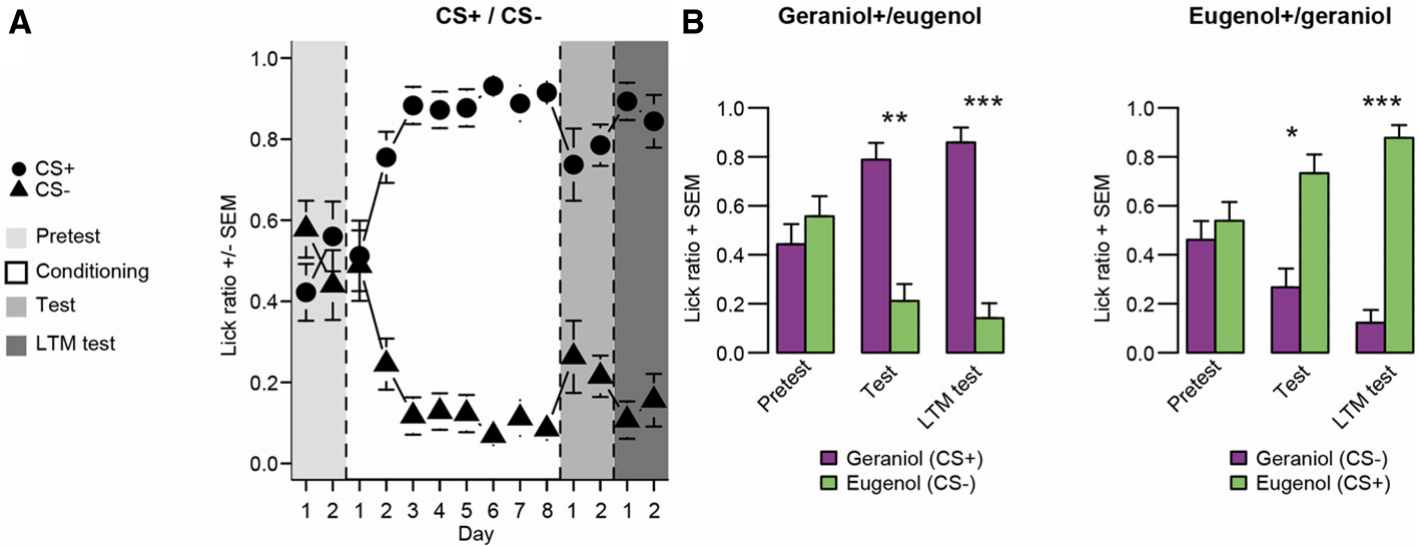
**Experiment 1: Tests for conditioned odor preference induced in the two groups of rats after conditioning and one month later. (A)** Learning curve and test followed by long-term memory test (LTM test) performances for the two groups pooled (*n* = 16). During the conditioning period, the CS+ odor was associated with the sucrose solution and the CS- with plain water. Before and after conditioning (pretest and tests), both odors were associated with plain water. **(B)** Odor preference for each group (left panel: group 1 conditioned for G+ over E−; right panel: group 2 conditioned for E+ over G−; *n* = 8 for each group). Data are presented as means of ratio (number of licks for the bottle on the sum of the number of licks for the two bottles) + s.e.m. over the two consecutive pretest/test days (Wilcoxon tests; ^*^p < 0.05; ^**^p < 0.01; ^***^p < 0.001).

Before conditioning, no significant preference between geraniol and eugenol was detected in any group (WT: group 1, *p* = 0.326; group 2, *p* = 0.552). However, as soon as the second odor-sucrose association was run, rats exhibited a clear preference for the odor paired with the sucrose solution. We observed that the odor was progressively used to rapidly locate the sucrose solution. After conditioning, both groups showed a preference for the bottle associated with the odor previously paired with sucrose (WT test: group 1, *n* = 8, *p* = 0.002; group 2, *n* = 8, *p* = 0.011, see Figure 2). When rats from both groups were tested again 1 month later, their preference for the odor previously paired with sucrose was still significant (WT: group1, *n* = 8 and group 2, *n* = 8, *p* = 0.001).

#### Discussion

Experiment 1 confirmed the efficiency of our conditioning protocol. Although no spontaneous preference was observed for geraniol or eugenol, as expected, in both groups, a clear preference for the sucrose-paired odor developed after 8 days of conditioning. This olfactory preference was independent of the odor paired with sucrose since the two groups had a similar learning curve. Interestingly, this acquired preference was stable since, even 1 month after the last odor-sucrose exposure; the rats still preferred the odor previously paired with sucrose.

### Experiment 2

#### Specific methods

Three pretests were conducted before conditioning to assess possible spontaneous preference. For that purpose, odors were presented in pairs both associated with water, on separate sessions and in the following order: carvone[+] (C[+]) vs. carvone[−] (C[−]), carvone[+] vs. limonene[−] (L[−]).

These pretests were followed by an 8-day period of conditioning. During this phase, carvone [−] was associated with sucrose (C[−]+suc) L[−] with water (L[−]−). After conditioning, we first presented C[−] vs. L[−] both associated with tap water to confirm the acquisition of odor preference. We then separated the sixteen rats into two groups (3 and 4) mixing animals from the two groups previously used (group 1 and group 2). In group 3, we tested C[+] vs. L[−] and then C[−] vs. C[+]. In group 4, we reversed the test order and first tested C[−] vs. C[+], and then C[+] vs. L[−] (see Table 1 for a summary of all performed tests).

#### Results (Figure 3)

Regarding the pretests performed before conditioning, no significant difference of intake between C[−] and C[+] (*n* = 16, WT, *p* = 0.940), C[+] and L[−] (*n* = 16, WT, *p* = 0.537) and C[−] and L[−] (*n* = 16, WT, *p* = 0.640) emerged from the pretests.

**Figure 3:**
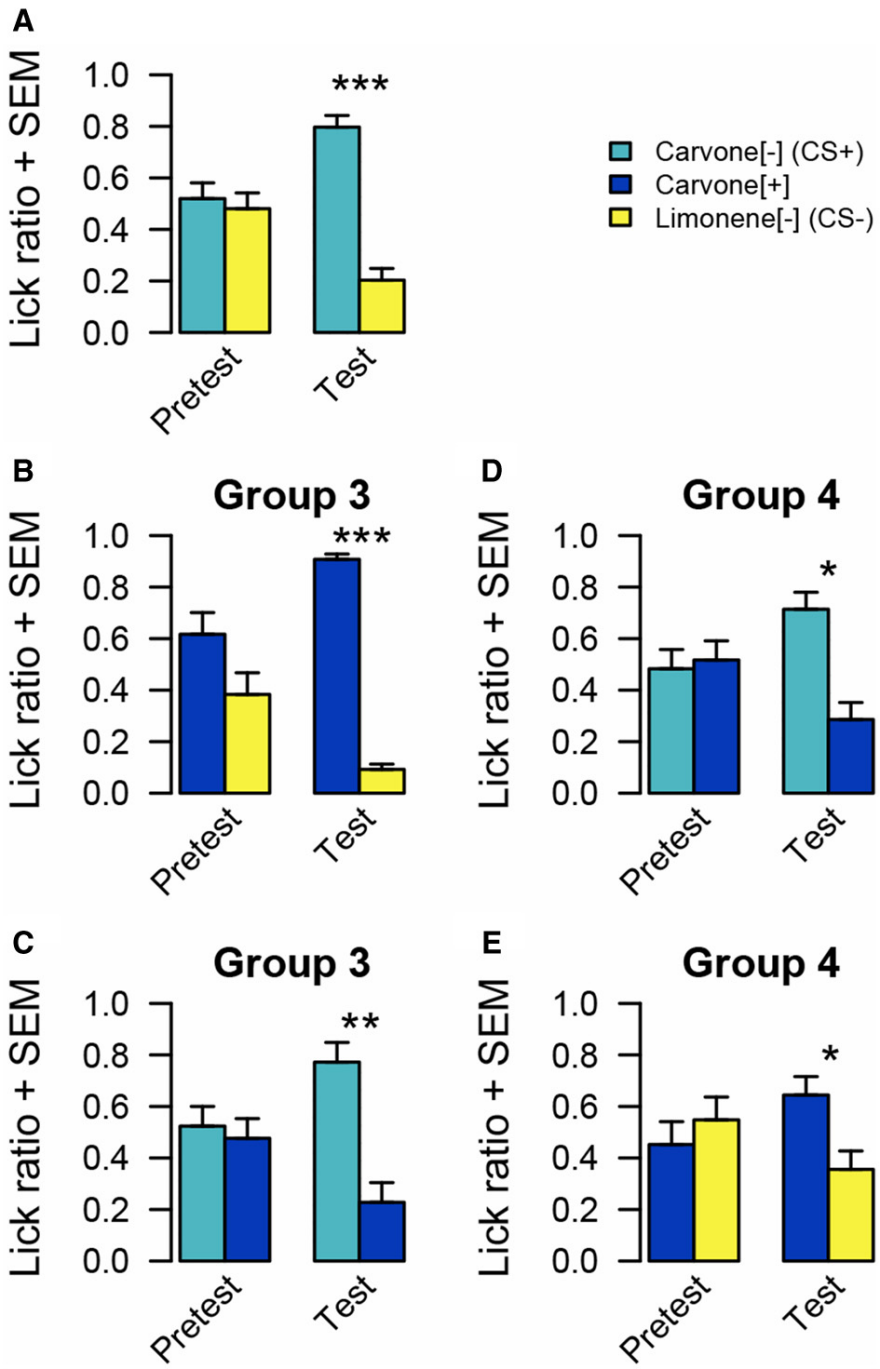
**Odor preference measured by lick ratio in experiment 2. (A)** For all rats, there is no preference between C[−] and L[−] before conditioning. After conditioning, animals preferred C[−]. **(B)** For group 3, there is no significant difference between C[+] and L[−] before conditioning. After conditioning, rats preferred C[+]. **(C)** For group 3, there is no preference before conditioning between C[−] and C[+]. After conditioning, animals preferred C[−]. **(D)** For group 4, there is no odor preference between C[−] and C[+] before conditioning. After conditioning, rats preferred C[−]. **(E)** Before conditioning, rats of group 4 have no preference between C[+] and L[−]. After conditioning, they preferred C[+]. Data are presented as means of ratio (number of licks for the bottle on the sum of the number of licks for the two bottles) + s.e.m over the two successive pretest/test days (Wilcoxon tests; ^*^p < 0.05; ^**^p < 0.01; ^***^p < 0.001).

After pairing sucrose to C[−], as expected, rats drank more from the bottle associated with this odor (*n* = 16, WT, *p* < 0.001) than L[−] even in the absence of sucrose. In the following tests, rats from group 3 preferred to drink from the bottle associated with C[+] rather than from the one associated with L[−] (*n* = 8, WT, *p* < 0.001). However, when C[+] was simultaneously presented with C[−] the rats exhibited a preference for the bottle odorized with the sucrose-paired odorant C[−] (*n* = 8, WT *p* = 0.009).

Rats from group 4 submitted to the same tests in a reverse order, drank more from the bottle associated with C[−] than from the one associated with C[+] (*n* = 8, WT *p* = 0.011). Subsequently, they also preferred C[+] to L[−] (*n* = 8, WT, *p* = 0.049).

#### Discussion

This second experiment confirmed the efficacy of the protocol to induce an odor preference with another pair of stimuli. This experiment also addressed the question of specificity for odor preference acquisition. This was assessed by adjusting carvone enantiomers discriminability. Before conditioning, both groups of rats exhibited no preference between these two odors. This result could be interpreted in two ways: either the animal is not able to discriminate these two odorant molecules or it is indeed able to discriminate them but has no preference. After conditioning, both groups exhibited a preference for the enantiomer previously paired with sucrose (C[−]) when simultaneously presented with L[−] or with C[+]. When a preference for C[+] to L[−] was observed, one could interpretate that the rats transferred the positive value acquired by one enantiomer to the other because they are perceived as similar. However, after conditioning, both groups exhibited a preference for C[−] vs. C[+], implying that the rats have the capacity to discriminate among the two carvone enantiomers. This change, compared to the pretest situation, could be interpreted either as a learning-induced improvement of enantiomer discrimination as suggested by others (Escanilla et al., 2008) and/or by a learning-induced change in motivation for choosing C[−]. A further hypothesis that fits with the previous two ideas, is that rats drinking more from the C[+] bottle when simultaneously presented with L[−], could be due to a generalized sugar association to “carvons” or simply because they avoided the odor that has never been paired with sucrose. This issue was addressed in experiment 3.

### Experiment 3

#### Specific methods

This experiment was carried out on a group of 24 rats. Four pretests were performed to evaluate spontaneous preference for each odor presented by pairs before conditioning in the following order: carvone[−] (C[−]) vs. geraniol (G) for all rats (*n* = 24); limonene[−] (L[−]) vs. iso-amylacetate (I) for group 1 (*n* = 12) and eugenol (E) vs. carvone[+] (C[+]) for group 2 (*n* = 12); carvone[−] vs. limonene[−] for all rats (*n* = 24). The experiment included a conditioning phase. During this 8-day period, carvone[−] was associated with sucrose (C[−]+suc), and limonene[−] with water (L[−]). The acquired preference for C[−] vs. L[−] was then tested. On following sessions, group 1 was tested with L[−] vs. I and group 2 with C[+] vs. E. Finally, all rats were tested with C[−] vs. G (see Table 1).

#### Results (Figure 4)

Before conditioning C[−] and L[−] were equally approached (*n* = 24, WT, *p* = 0.082). Following conditioning, both groups acquired a clear preference for C[−] (*n* = 24, WT, *p* < 0.001).

**Figure 4:**
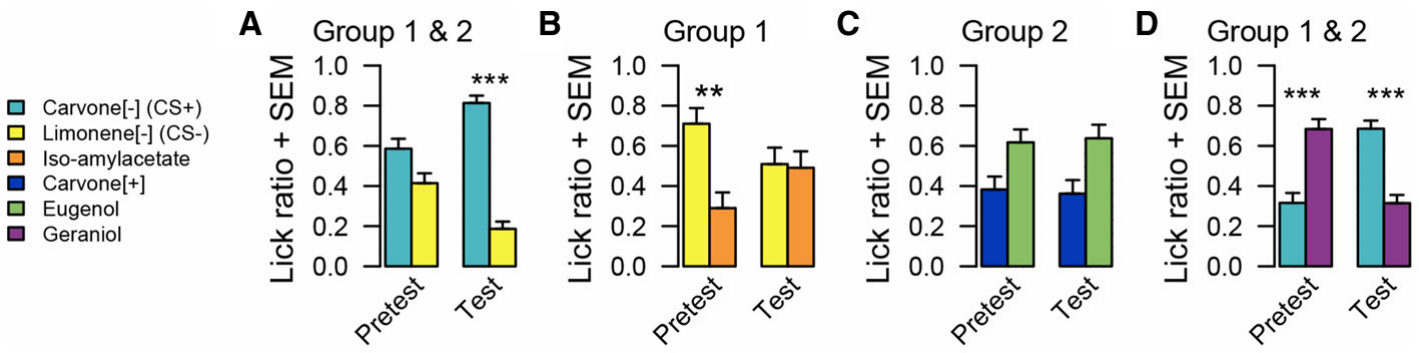
**Odor preference measured by lick ratio in experiment 3**. **(A)** For all animals, there is no preference between C[−] and L[−] before conditioning. After conditioning, animals preferred C[−]. **(B)** Animals of group 1 preferred L[−] over I before conditioning. After conditioning, this spontaneous preference disappeared. **(C)** There is no preference for animals of group 2 between C[+] and E before and after conditioning. **(D)** All animals preferred G over C[−] before conditioning. After conditioning, preference changed and animals preferred C[−] over G. Data are presented as means of ratio (number of licks for the bottle on the sum of the number of licks for the two bottles) + s.e.m. over the two successive pretest/test days (Wilcoxon tests; ^*^p < 0.05; ^**^p < 0.01; ^***^p < 0.001).

During the pretest, rats from group 1 exhibited a spontaneous preference for L[−] compared to I (*n* = 12, WT, *p* = 0.009), which was abolished during conditioning (*n* = 12, WT, *p* = 0.909). Rats from group 2 showed a tendency to prefer E over C[+] during the pretest but this failed to reach significance (*n* = 12, WT, *p* = 0.076), which was not modified after conditioning (*n* = 12, WT, *p* = 0.056). Pre-tests revealed a significant spontaneous preference for G vs. C[−] (*n* = 24, WT, *p* < 0.001). Conditioning reversed this preference (*n* = 24, WT, *p* < 0.001).

#### Discussion

The aim of experiment 3 was to elucidate the nature of change in the reward value induced by an odor preference conditioning. Thus, after preference acquisition for C[−] compared to L[−], each odor of this pair was presented with a novel one to evaluate the new preferences.

Before learning, animals from group 1 preferred to drink from the bottle associated with L[−] when this odor was simultaneously presented with I. This preference disappeared after conditioning, which led us to the conclusion that, during preference conditioning, rats also learnt that L[−] had never been associated with sucrose. As a consequence, its natural reward value decreased.

Moreover, results obtained from group 2 suggested that learning-induced changes in the reward value are specific to odorants used during the conditioning phase. Indeed, pairing C[−] with sucrose had no effect on how C[+] was perceived compared to E. We therefore interpret that in experiment 2, the generalization from C[−] to C[+] might be the consequence of avoidance of L[−] that has never been paired with sucrose.

Rats spontaneously preferred G to C[−], but after conditioning, their preference was reversed. This clearly indicates that learning increases carvone reward value. Putting together this result with those of the tests with C[+] and E, we could conclude that the present olfactory preference conditioning procedure selectively increased C[−] reward value and, as a consequence, devaluated L[−]. This experiment also confirmed the ability of rats to distinguish these two enantiomers, even when they had never been presented simultaneously for direct olfactory comparison.

## General discussion and conclusions

These series of experiments confirm the possibility to induce a strong and reliable conditioning olfactory preference by associating the consumption of a sucrose solution and the close delivery of an odor without any ingestion of it. Indeed, the odor concentrated around the water bottle's spout was perceived as a characteristic of the solution contained inside the bottle. Experiments 1 and 2 assessed, for the first time, a display of olfactory preference for two different odor pairs (eugenol, geraniol and carvone[−], limonene[−]). Conditioning led to a specific preference for the odor associated with a sucrose solution, and as shown in experiment 1, this preference was maintained for at least 1 month. Experiment 2 and 3 addressed the question of specificity for odor preference acquisition and elucidated the nature of change in the reward value induced by such an odor preference conditioning.

As mentioned in the introduction, few studies have explored the individual contribution of odor and taste in conditioned food preferences. In the experiment performed by Holder (1991), each odor was paired with a different sweet taste solution (either sucrose or saccharin) and the animals were continuously exposed during 4 days in their home cage and tested 48 h later, after a period of access to plain water. In our protocol, only one of the two odors was associated with a sugar solution while the other was paired with plain water. This presentation was performed in an experimental cage during 8 consecutive daily sessions of 15 min. Moreover, the animals were also allowed to drink water in their home cage for 20 min each day. The advantage of using such an exposure mode was to maximally control the experimental environment and to avoid any diffusion of the odorant and subsequent contextual learning. Despite these differences, in both experiments, a clear preference was obtained for the odor paired with sucrose compared to saccharin in Holder's study and water in our case. Sucrose solution has very often been used for appetitive conditioning (Holder, 1991; Boakes et al., 2007) since its sugar taste is highly and spontaneously preferred (Shepherd, 2006), with a non-negligible energy supply (Myers and Sclafani, 2001). Other protocols that have used saccharin to avoid energy supply (Baker and Booth, 1989; Myers and Sclafani, 2001); demonstrated that conditioned odor preferences were reinforced by the postingestive effects of caloric substances (Myers and Sclafani, 2001). Nevertheless this reinforcement has been suggested to be dependent on the feeding state of animals (Harris et al., 2000). We cannot rule out the possibility that the metabolic effect of sucrose has contributed to the success and stability of our conditioning procedure. However, the main objective of our study was not to address this specific issue but rather to increase as much as possible the rewarding value of the odor paired with sucrose.

In experiments 2 and 3, the strategy of the animal's choice was investigated. In experiment 2, the reinforced odor (C[−]+) was replaced by its enantiomer (C[+]) during the test and presented simultaneously with the non-reinforced odor (L[−]−). Rats seemed to consider the new odor as the previous reinforced one. This result could lead to two distinct interpretations, either the rats were unable to discriminate between the two enantiomers of carvone, or they were generalizing the olfactory preference to closely related odorants. However, when rats later had the choice between the two enantiomers, they showed that they could discriminate between them and correctly chose the reinforced odor (C[−]+). This result confirmed their ability to discriminate the enantiomers carvone[−]/carvone[+] when necessary and to generalize when appropriate. In this experiment, the order of the pair presentations was reversed for group 2. When the rats had to choose between the enantiomers (carvone[−]/carvone[+]) before choosing between C[+] and L[−]−. The group exhibited a good discrimination between the enantiomers but always chose C[+] vs. L[−]−. However, this preference was less marked, as if rats knew C[+] was not the previously reinforced odor. This last result triggered another question: why did the rats choose C[+] when compared with L[−]−? It could be due to a generalization of the acquired value of C[−]+ to “carvons,” or to a decrease in the reward value of the non-reinforced odor (L[−]−). Experiment 3 allowed us to answer this question and to validate the second hypothesis.

**Figure 5:**
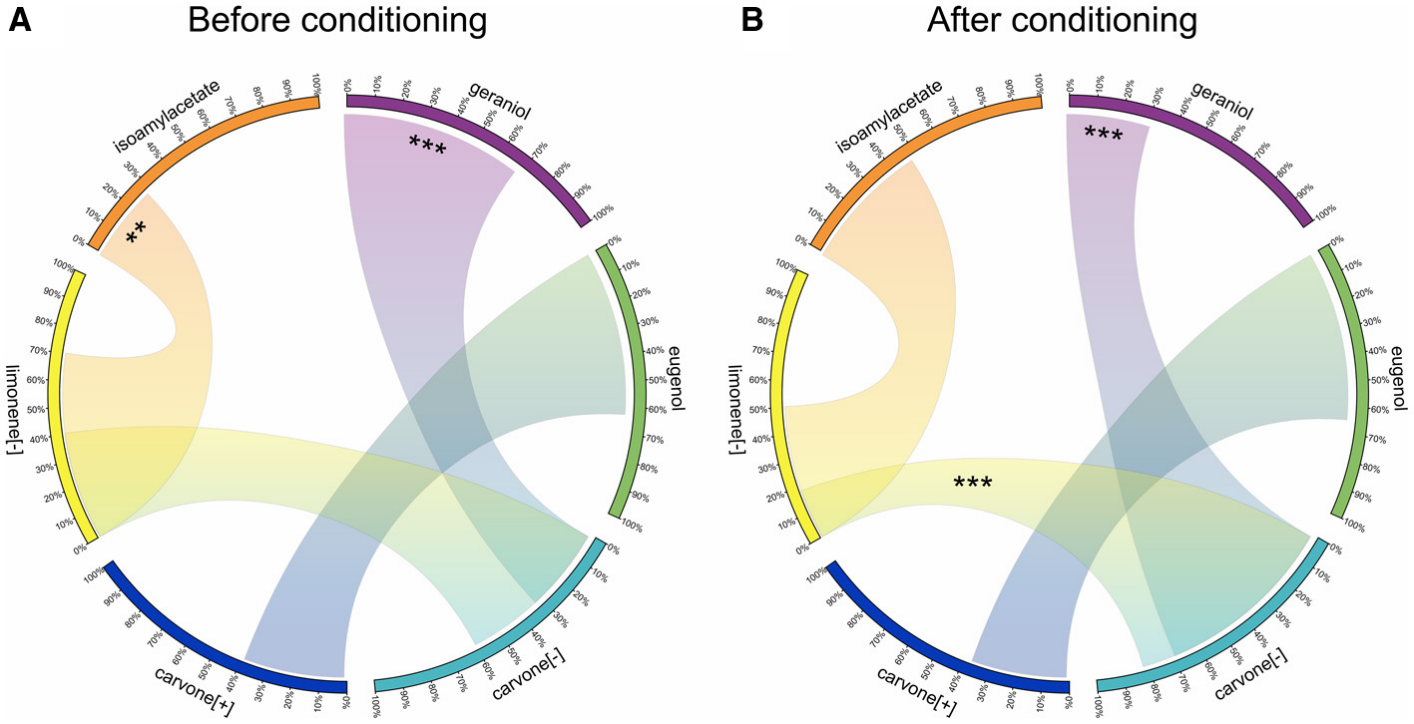
**Variations of preference for 6 odors in experiment 3, suggesting changes in the reward value after conditioning**. Each odor used in experiment 3 is represented on a portion of the circle. On each of these portions, a scale represents the proportion of licks. Each portion of circle (i.e., each odor) is connected to another one to compare the preference (indicated by the proportion of licks) between these two odors: the larger the link is, the more preferred the odor is. **(A)** Spontaneous odor preference before conditioning during the pretests. **(B)** Odor preference after conditioning with reinforcement on C[−] during the tests. Data are presented as means of ratio (number of licks for the bottle on the sum of the number of licks for the two bottles) over the two successive pretests/test days (Wilcoxon tests; ^*^p < 0.05; ^**^p < 0.01; ^***^p < 0.001).

The goal of experiment 3 was indeed to test whether COP also modified the reward value of the non-reinforced odor. This was assessed by comparing how the conditioning procedure affected the preference between the odor paired with water in our paradigm and different odors never included in the conditioning procedure. Figure 5 summarizes the variations of reward value for the odors used in experiment 3, following pairing C[−] with sucrose and L[−] with water. The comparison of preference before (Figure 5A) and after (Figure 5B) the olfactory conditioning shows that, as expected, C[−] became preferred over L[−]. In addition, the initial preference for G vs. C[−] was reversed by the conditioning. This confirmed a development of preference for the odor paired with sucrose during conditioning, independently of the value of L[−]. In experiment 2, rats preferentially chose C[+] over L[−]. We first hypothesized that this could be explained by a generalization between the two enantiomers of carvone. However, the initial spontaneous preference for L[−] vs. I disappeared totally, suggesting a decrease in the reward value of L[−] through conditioning. For new pairs of odors like E vs. C[+], conditioning had no influence on the preference for one or the other odor. If there was a generalization between the two carvones, C[+] would have been preferred over E as was the case for C[−]+ compared to G. This comparison therefore weakens the hypothesis of a generalization between the two carvones and instead confirms a decrease in reward value for L[−]− after conditioning, leading to a preference for C[+] when the two odors were simultaneously proposed.

In the present protocol, changes in reward value were measured by the comparison of the consumption of two solutions, each associated with a different odor. Conditioning resulted in a preference for one of these odors, but, interestingly, the non-reinforced odor was also avoided when challenged with another odor. It remains an open question as to whether this avoidance reflects a strategy (a kind of learning by exclusion within the comparison of the two odors) or whether it reflects a real change in reward value, with a depreciation of the value of this odor (i.e., this odor acquires a negative value). Indeed, two theories could explain this reduced consumption of water associated with L[−] after conditioning. The first one is the *model-free theory*. In this case, animals learn by trial-and-error and each new trial reinforces their preference for the sucrose-associated odor (Doll et al., 2012). According to this theory, after many comparisons between C[−]+ and L[−]−, rats consider C[−]+ as rewarding and they will actively seek it. This theory interprets the learnt avoidance of L[−]− as reflecting a negative value, in a kind of habit behavior acquired during conditioning. However, it seems unlikely that water alone decreases the value of the odor with which it is associated. The *model-based theory*, in contrast with the model-free learning, describes adaptive and dynamic value inference in learning tasks (using a “world model”) (Doll et al., 2012), where the animal learns the structure of the task. For instance, it infers that the structure of the test is similar to the conditioning, that is, the test always includes a non-reinforced odor and a reinforced one. In this perspective, L[−]− would not become a negative cue, and would not be avoided, but would signal the other odor to be potentially reinforced, explaining the preference. Furthermore, decisions would arise from a goal-directed control, in contrast to the habit behavior developed in the model-free theory. An interesting insight on these processes may come from the extinction of this preference and, more precisely, from the observation of potential changes in reward values over time, for the CS+ and CS−, respectively.

The present protocol offers a framework to explore in parallel the behavior and the neurobiological processes involved in olfactory appetitive learning. Indeed, in this paradigm the odors are always perceived in an orthonasal way and odor-taste integration might have recruited circuits distinct from those of flavor perception. Using an olfactory aversion paradigm, our group described two different circuits involved according to how the odor was presented (ortho vs retronasally) to the animal during conditioning (Chapuis et al., 2009). It would be interesting to test whether if it is also the case for appetitive learning. More generally, trying to understand the importance of smell to the perception of flavor and the formation of cognitive and emotional responses to food will contribute to the biomedical knowledge required to solve today's rising obesity and diabetes rates.

### Conflict of interest statement

The authors declare that the research was conducted in the absence of any commercial or financial relationships that could be construed as a potential conflict of interest.
